# Dietary fiber intake and glycemic control: coronary artery calcification in type 1 diabetes (CACTI) study

**DOI:** 10.1186/s12937-019-0449-z

**Published:** 2019-04-03

**Authors:** Arpita Basu, Amy C. Alman, Janet K. Snell-Bergeon

**Affiliations:** 1 Epidemiology and Biostatistics, University of South Florida, Tampa, USA; 2 Department of Kinesiology and Nutrition Sciences, University of Nevada Las Vegas, Las Vegas, USA; 3 Barbara Davis Center for Childhood Diabetes, University of Colorado, Anschutz Medical Campus, Aurora, USA

**Keywords:** Dietary fiber, Hemoglobin A1c, Type 1 diabetes, Glycemia

## Abstract

**Background:**

Dietary fiber has been recommended for glucose control, and typically low intakes are observed in the general population. The role of fiber in glycemic control in reported literature is inconsistent and few reports are available in populations with type 1 diabetes (T1D).

**Methods:**

Using data from the Coronary Artery Calcification in Type 1 Diabetes (CACTI) study [*n* = 1257; T1D: *n* = 568; non-diabetic controls: *n* = 689] collected between March 2000 and April 2002, we examined cross-sectional (baseline) and longitudinal (six-year follow-up in 2006–2008) associations of dietary fiber and HbA1c. Participants completed a validated food frequency questionnaire, and a physical examination and fasting biochemical analyses (12 h fast) at baseline visit and at the year 6 visit. We used a linear regression model stratified by diabetes status, and adjusted for age, sex and total calories, and diabetes duration in the T1D group. We also examined correlations of dietary fiber with HbA1c.

**Results:**

Baseline dietary fiber intake and serum HbA1c in the T1D group were 16 g [median (IQ): 11–22 g) and 7.9 ± 1.3% mean (SD), respectively, and in the non-diabetic controls were 15 g [median (IQ): 11–21 g) and 5.4 ± 0.4%, respectively. Pearson partial correlation coefficients revealed a significant but weak inverse association of total dietary fiber with HbA1c when adjusted for age, sex, diabetes status and total calories (r = − 0.07, *p* = 0.01). In the adjusted linear regression model at baseline, total dietary fiber revealed a significant inverse association with HbA1c in the T1D group [β ± SE = − 0.32 ± 0.15, *p* = 0.034], as well as in the non-diabetic controls [− 0.10 ± 0.04, *p* = 0.009]. However, these results were attenuated after adjustment for dietary carbohydrates, fats and proteins, or for cholesterol and triglycerides. No such significance was observed at the year 6 follow-up, and with the HbA1c changes over 6 years.

**Conclusion:**

Thus, at observed levels of intake, total dietary fiber reveals modest inverse associations with poor glycemic control. Future studies must further investigate the role of overall dietary quality adjusting for fiber-rich foods in T1D management.

## Background

The incidence of type 1 diabetes (T1D), an autoimmune disorder, as well as cardiovascular disease (CVD), the major vascular complication of diabetes have been increasing worldwide [1, 2]. Based on the statistics reported by the American Heart Association, only 1.5% of US adults meet the guidelines for healthy diet pattern [2]. While the recommendation of healthy dietary pattern may have poor compliance in populations, the role of modifying individual nutrients and bioactive compounds has gained much attention in the management of chronic diseases such as T1D. Among the dietary components, fiber has been shown to play an important role in glycemic control in diabetes [3]. In a meta-analysis of randomized clinical trials reported by Silva et al. (2013), higher fiber diets (up to 42.5 g/day) or supplements containing soluble fiber (15 g/day) were found to significantly decrease HbA1c and fasting plasma glucose in adults with type 2 diabetes [4]. In another systematic review, foods rich in soluble fiber, such as beta-glucans, were shown to improve glycemia in diabetes patients [5]. These effects of fiber have been explained by biological mechanisms in delaying gastric emptying and decreasing glucose absorption that subsequently lead to decreases in postprandial rise of blood glucose [6, 7]. In addition, dietary fiber, especially increased soluble fiber intake, has also been associated with anti-inflammatory properties and in modulating the immune system with potential implications in the prevention of T1D in children [8].

Epidemiological evidence on the associations of dietary fiber and glycemic control in adults with type 1 diabetes is limited and conflicting. In a longitudinal study of youths with type 1 diabetes (*n* = 136), following behavioral nutrition intervention, fiber intake was associated with improved glycemic control [9]. Data from the European Diabetes Centers (EURODIAB) Prospective Complications Study have reported lower fiber intake in adults with type 1 diabetes (*n* = 1102) [10], as well as an inverse association of fiber with HbA1c in these adults (*n* = 1659) [11]. In another study, fiber intake was not associated with HbA1c control in youths (*n* = 908) in a longitudinal dietary study [12]. While several factors may be involved in the differences in these study findings, participant characteristics, such as levels of dietary fiber intake and cardio-metabolic profiles, and duration of diabetes may play an important role, and thus a need to further investigate these associations in well-defined cohorts of T1D.

We have previously reported low prevalence of ideal cardiovascular health (1.1%), especially based on the American Heart Association definition of health matrices, in adults with type 1 diabetes in the coronary artery calcification in type 1 diabetes (CACTI) study [13]. We now aim to identify the associations of dietary fiber with glycemic control in the same cohort at cross-sectional and longitudinal time points.

## Methods

### Study participants

The data presented in this report were collected as part of the baseline examination of the CACTI study. The study enrolled 1416 individuals between 19 and 56 years of age, with no known history of CHD: 652 subjects with type 1 diabetes and 764 nondiabetic control subjects. Participants with type 1 diabetes had long-standing disease (mean duration 23 years, range 4–52 years), were insulin dependent within a year of diagnosis, and were diagnosed prior to age 30 or had positive antibodies or a clinical course consistent with type 1 diabetes. Non-diabetic control subjects had fasting blood glucose < 110 mg/dL and were generally spouses, friends and neighbors of cases. The inclusion and exclusion criteria have been described previously [14]. All study participants provided informed consent and the study protocol was approved by the Colorado Multiple Institutional Review Board.

### Dietary intake

Study participants who completed the baseline screening visit were asked to fill out a validated self-administered semi quantitative food-frequency questionnaire of 126 food items (Harvard FFQ, 1988) [15]. The procedures have been previously published in detail [14]. The nutrient values and dietary fiber content of foods for the Harvard FFQ were estimated primarily on the basis of the USDA food composition database, and the correlation coefficient for dietary fiber was 0.68 based on the validation studies [15, 16]. The total dietary fiber content was mainly derived from the following food groups included in the FFQ: breakfast cereals, breads, other cereal foods, potatoes, legumes, lentils, vegetables, fruits, and nuts and seeds. One thousand three hundred six study participants completed the FFQ. Calorie intake was calculated per the guidelines suggested by Willett [17] for excluding individuals with implausible reported energy intake, 40 participants were excluded due to reported caloric intake that was very low (< 500 cal per day if female or < 800 cal per day if male), or very high (3500 or higher if female, 4000 or higher if male), leaving 571 participants with type 1 diabetes and 696 controls in this analysis [14]. Among these, for the current analysis, complete biochemical and dietary information was available in 568 participants with type 1 diabetes and 689 controls and were included in the final cross-sectional analysis.

### Cardiovascular risk factors

Participants completed a baseline examination between March 2000 and April 2002. Anthropometric measurements were obtained and included height, weight and waist circumference. Body mass index was calculated in kg/m^2^. Resting systolic blood pressure (SBP) and fifth-phase diastolic blood pressure (DBP) were measured three times while the patients were seated, following a 5 min rest, and the second and third measurements were averaged (Omron HEM-705CP). In addition, participants completed standardized questionnaires that enquired about medical history, current medication, insulin doses, physical activity, alcohol and tobacco use and family medical history. Following a 12 h fast, participants came to the clinic in the morning for blood collection and analyses of biochemical variables. Lipids (total cholesterol, HDL-cholesterol, LDL-cholesterol and triglycerides), fasting glucose and HbA1c were measured. After an overnight fast, blood was collected, centrifuged and separated. Plasma was stored at 4 °C until assayed. Total plasma cholesterol and triglyceride levels were measured using standard enzymatic methods; HDL cholesterol was separated using dextran sulphate, and LDL cholesterol was calculated using the Friedewald formula. High-performance liquid chromatography was used to measure HbA_1c_ (HPLC, BioRad variant).

### Statistical analysis

Variables were examined for normality (normal plots), and non-normally distributed variables (dietary fiber, plasma triglycerides) were log transformed. Differences in risk factors between men and women with type 1 diabetes and without diabetes were examined using a Student’s t test. A *χ*2 test for goodness of fit was used to determine if categorical risk factors differed between patients with type 1 diabetes and non-diabetic participants. Wilcoxon rank sum test was used to compare differences of continuous variables with skewed distributions. Correlations of dietary fiber with HbA1c, and dietary macronutrients (carbohydrates, total fats and proteins as percentage of daily caloric intake) and cardiovascular risk factors (systolic and diastolic blood pressure, BMI, waist circumference, plasma total-, HDL- and LDL-cholesterol and triglycerides, and glucose) were examined using Pearson correlation coefficients after adjusting for age, sex, diabetes status and total calories in the entire cohort, as well as by T1D status. Linear regression analysis was used to examine associations of dietary fiber intake at baseline with contemporaneous HbA1c, as well as with HbA1c at year 6 follow-up and the change between year 6 and baseline. Models were adjusted for relevant covariates as follows: model 1 (age, sex, and total calories, and diabetes duration for T1D), model 2 (model 1 + dietary carbohydrates, fats and proteins) and model 3 (model 1 + plasma lipids). In addition, longitudinal analyses were adjusted for baseline HbA1c and the duration of follow-up. Logistic regression analysis was used to examine associations of quintiles of total dietary fiber with the probability of poor control (> 7%) vs. optimal control (< 7%) of HbA1c at baseline and the 6-year follow-up.

## Results

A total of 1257 participants were included in the cross-sectional analysis, and a total of 990 participants who had HbA1c values at both baseline and Year 3 were included in the longitudinal analysis in this report. Table 1 shows the baseline characteristics of the study participants stratified by sex and diabetes status. Women were younger than men in both groups, significantly so in the non-diabetic control group. Among the anthropometric and biochemical measures in the T1D group, BMI, waist circumference, systolic and diastolic blood pressure were significantly lower in women, while HDL-cholesterol was higher when compared to men. We observed similar differences in the control group, in addition to significantly lower fasting glucose, total cholesterol and triglycerides in women than in men. Among the dietary nutrient intakes, total energy intake was significantly lower, while carbohydrate and protein intake were modestly higher in women than men in both groups. No significant differences in total fiber intake were observed between men and women in the T1D or control group (Table 1).

**Table 1:** Baseline characteristics of the CACTI cohort

	Type 1 diabetes			Non-diabetic control		
	Men (*n* = 251)	Women (*n* = 320)	*p*-value	Men (*n* = 347)	Women (*n* = 349)	*p*-value
Age (years)	38.0 ± 9.0	36.0 ± 9.0	0.07	40.0 ± 9.0	38.0 ± 9.0	**0.01**
HbA1c (%)	7.9 ± 1.2	7.9 ± 1.3	0.75	5.6 ± 0.4	5.4 ± 0.4	**< 0.0001**
Met < 7% HbA1c goal (%)	18.7	22.0	0.67	N/A	N/A	N/A
Duration of diabetes (years)	24.0 ± 9.0	23.0 ± 9.0	0.18	N/A	N/A	N/A
Duration of follow-up (years)	6.2 ± 0.6	6.2 ± 0.5	0.23	6.1 ± 0.5	6.2 ± 0.6	0.25
BMI (kg/m^2^)	26.6 ± 3.8	25.8 ± 4.7	**0.04**	27.2 ± 4.1	25.1 ± 5.6	**< 0.0001**
Waist circumference (cm)	90.8 ± 11.0	81.0 ± 12.0	**< 0.0001**	93.0 ± 12.0	79.0 ± 13.0	**< 0.0001**
Systolic blood pressure (mm Hg)	122.0 ± 13.0	114.0 ± 14.0	**< 0.0001**	118.0 ± 11.0	111.0 ± 13.0	**< 0.0001**
Diastolic blood pressure (mm Hg)	80.0 ± 9.0	75.0 ± 8.0	**< 0.0001**	82.0 ± 8.0	76.0 ± 8.0	**< 0.0001**
Plasma glucose (mg/dL)	199.0 ± 102.0	187.0 ± 93.0	0.14	93.0 ± 10.0	87.0 ± 9.0	**< 0.0001**
Plasma cholesterol (mg/dL)	175.0 ± 35.0	176.0 ± 33.0	0.81	198.0 ± 43.0	185.0 ± 34.0	**< 0.0001**
Plasma HDL-cholesterol (mg/dL)	50.0 ± 13.0	60.0 ± 17.0	**< 0.0001**	43.0 ± 11.0	58.0 ± 14.0	**< 0.0001**
Plasma triglycerides (mg/dL)	83.0 (62.0–114.0)	77.0 (62.0–103.0)	0.08	123.0 (89.0–181.0)	89.0 (66.0–124.0)	**< 0.0001**
Energy intake (kcal/day)	1954.0 ± 625.0	1633.0 ± 561.0	**< 0.0001**	1992.0 ± 655.0	1655.0 ± 528.0	**< 0.0001**
Carbohydrate intake (% kcal/day)	44.0 (38.0–51.0)	46.0 (40.0–52.0)	**0.04**	47.0 (42.0–52.0)	48.0 (42.0–54.0)	**0.04**
Fat intake (% kcal/day)	36.0 (31.0–41.0)	35.0 (30.0–39.0)	0.08	34.0 (29.0–37.0)	33.0 (28.0–36.0)	0.06
Protein intake (% kcal/day)	18.0 (16.0–21.0)	19.0 (17.0–21.0)	**0.004**	18.0 (15.0–20.0)	19.0 (16.0–21.0)	**0.0004**
Total dietary fiber (g)	16.0 (12.0–22.0)	15.0 (11.0–21.0)	0.06	15.0 (11.0–21.0)	16.0 (11.0–21.0)	0.88
Physical activity (kJ/week)	7517 (3165–14,118)	5020 (1938–10,862)	0.32	6986 (3048–12,861)	6232 (2859–11,255)	0.21

Data are presented as means ± SD and median (IQ range)

*P* < 0.05 in bold; Comparison between men and women: t test for difference in means, χ2 test for difference in proportions, and Wilcoxon rank sum test for difference of continuous variables with skewed distributions

Table 2 shows adjusted correlation coefficients of dietary fiber intake with anthropometrics, biochemical and dietary variables in the entire cohort, as well as by diabetes status. Dietary fiber exhibited a significant and inverse correlation with HbA1c, BMI, waist circumference, systolic and diastolic blood pressure, serum cholesterol and triglyceride. Among the dietary nutrients, fiber intake revealed a significant positive association with total carbohydrates, and an inverse association with total fat intake in the entire cohort (all *p* < 0.05). These significant correlations persisted in non-diabetic controls, but were somewhat attenuated in T1D cases for BMI, waist circumference, plasma HDL-C, triglycerides and glucose. Overall, total dietary fiber intake remained inversely correlated with HbA1c in T1D cases as well as non-diabetic controls (Table 2).

**Table 2:** Pearson partial correlation coefficients of total dietary fiber with clinical parameters and dietary variables in the CACTI cohort

Variable	Total dietary fiber (all subjects) (*n* = 1257)		Total dietary Fiber T1D cases (*n* = 568)		Total dietary fiber non-diabetic controls (*n* = 689)	
	R	*p*-value	R	*p*-value	R	*p*-value
HbA1c	−0.07	**0.01**	−0.08	**0.03**	− 0.10	**0.009**
Systolic blood pressure	−0.11	**0.0001**	−0.08	0.05	−0.12	**0.002**
Diastolic blood pressure	−0.13	**< 0.0001**	−0.09	**0.03**	−0.13	**0.001**
BMI	−0.14	**< 0.0001**	−0.02	0.56	−0.16	**< 0.0001**
Waist circumference	−0.12	**< 0.0001**	−0.01	0.73	−0.14	**0.0003**
Plasma cholesterol	−0.09	**0.0007**	−0.12	**0.005**	−0.06	0.11
Plasma HDL- cholesterol	0.04	0.16	−0.02	0.56	0.11	**0.004**
Plasma triglyceride	−0.07	**0.009**	−0.008	0.85	−0.10	**0.007**
Plasma glucose	−0.03	0.33	−0.02	0.59	−0.08	**0.022**
Carbohydrate (% daily intake)	0.43	**< 0.0001**	0.45	**< 0.0001**	0.43	**< 0.0001**
Protein intake (% daily intake)	−0.05	0.089	−0.08	0.05	−0.04	0.26
Fat intake (% daily intake)	−0.41	**< 0.0001**	−0.40	**< 0.0001**	− 0.43	**< 0.0001**

Based on log transformed values of dietary fiber

Adjusted for age, sex, total calories and diabetes status, and duration (T1D)

*T1D* type 1 diabetes

*P* < 0.05 in bold

Table 3 shows the associations of baseline dietary total fiber intake as a continuous variable with HbA1c at year 6 and the change (year 6 – baseline) stratified by diabetes status. Cross-sectional analysis at baseline revealed a significant inverse association of dietary fiber intake with HbA1c in the model adjusted for age, sex, total calories and diabetes duration in the T1D group, as well as in non-diabetic controls (Model 1). The significance did not persist in models further adjusted for dietary nutrients (Model 2) and conventional lipids (Model 3). Longitudinal analyses revealed no significant association in any of the models examined. The six-year change in HbA1c was observed to be 0.05 (− 0.69–0.63) %, median (IQR) in the T1D group, and 0.01 (− 0.2–0.30) %, in the non-diabetic controls. Log-transformed values of fiber were used for analyses presented in Tables 2 and 3.

**Table 3:** Linear associations of total dietary fiber with HbA1c at baseline, prospective (year 6) and change data in the CACTI cohort

Dietary fiber (Baseline)	HbA1c (Baseline)				HbA1c (year 6)				HbA1c change (year 6-Baseline)			
	T1D (*n* = 568)		Non-diabetic control (*n* = 689)		T1D (*n* = 452)		Non-diabetic control (*n* = 538)		T1D (*n* = 452)		Non-diabetic control (*n* = 538)	
	beta±SE	*P*-value	beta±SE	*P*-value	beta±SE	*P*-value	beta±SE	*P*-value	beta±SE	*P*-value	beta±SE	*P*-value
Model 1^a^	− 0.32 ± 0.15	**0.034**	**−**0.10 ± 0.04	**0.009**	0.017±0.138	0.90	0.033±0.057	0.56	0.017 ± 0.138	0.90	0.033 ± 0.057	0.56
Model 2^b^	**−** 0.14 ± 0.18	0.43	−0.06 ± 0.05	0.27	0.112±0.158	0.48	0.044±0.065	0.50	0.112±0.158	0.48	0.044±0.065	0.50
Model 3^c^	− 0.08 ± 0.11	0.46	−0.05 ± 0.03	0.19	0.119±0.158	0.45	0.058±0.065	0.37	0.119±0.158	0.45	0.058±0.065	0.37

*T1D* type 1 diabetes

*P* < 0.05 are in bold font

^a^Model adjusted for age, sex, diabetes duration and total calories for T1D; age, sex and total calories for non-diabetic control; year 6 and change model also adjusted for baseline HbA1c and duration of follow-up

^b^Model 1+ dietary carbohydrates, fats and proteins

^c^Model 1 + plasma total cholesterol and triglycerides

Table 4 shows data from logistic regression analysis to examine the associations of quintiles of dietary fiber intake with the probability of poor vs. optimal glycemic control (HbA1c > 7% vs. < 7%). At baseline, no significant associations were noted, but those in the highest category of fiber intake [29 (23-69 g), mean (range)] compared to the lowest (reference), trended towards an inverse association in adjusted analysis (*p* = 0.08, Table 4). No significant associations were noted at year 6 follow-up. No significant interaction effects of dietary fiber and sex were noted in our analyses (*p* = 0.22).

**Table 4:** Logistic regression associations of quintiles of total dietary fiber with the probability of poor control (> 7%) vs. optimal control (< 7%) HbA1c at baseline and at year 6 of CACTI study

Dietary fiber quintiles (g)	HbA1c goals (baseline) (> 7% *N* = 453 vs. < 7% *N* = 115)		HbA1c goals (year 6) (> 7% *N* = 361 vs. < 7% *N* = 91)	
	OR (95% CI)	*P*-value	OR (95% CI)	*P*-value
Quintile 1	Reference		Reference	
Quintile 2	0.487 (0.147, 1.617)	0.24	1.197 (0.546, 2.625)	0.65
Quintile 3	0.420 (0.126, 1.407)	0.16	1.059 (0.475, 2.359)	0.88
Quintile 4	0.370 (0.096, 1.423)	0.15	1.987 (0.787, 5.021)	0.15
Quintile 5	0.283 (0.07, 1.153)	0.08	1.573 (0.559, 4.432)	0.39

Data presented as OR (95% CI)

Dietary fiber quintiles: Quintile 1[N: 253; mean: 7.8 g (range: 2.8–10.3 g)]; Quintile 2 [N: 254; mean: 12.0 g (range: 10.3–13.7 g)]; Quintile 3 [N: 253; mean: 15.5 g (range: 13.7–17.4 g)]; Quintile 4 [N: 254; mean: 19.8 g (range: 17.4–22.7 g)]; Quintile 5 [N: 253; mean: 28.8 g (range: 22.7–68.5 g)]

Model adjusted for age, sex, diabetes duration and total calories; year 6 also adjusted for baseline HbA1c and duration of follow up

## Discussion

To our knowledge few reports have been published on the association between dietary fiber intake and glycemic control in T1D. Thus, our study found a significant inverse association between total dietary fiber intake and HbA1c levels at the baseline visit of the CACTI study in those with diabetes, as well as in non-diabetic controls in a model adjusted for standard covariates including total calories. Our significant cross-sectional associations of fiber with glycemic control did not persist in models further adjusted for dietary macronutrients and blood lipids, largely due to their known independent associations with glycemic control [18, 19]. We did not observe any significant predictive association of baseline fiber intake with HbA1c levels at year 6, and the six-year changes of HbA1c in adjusted models. These discrepancies in observations between cross-sectional and prospective associations may be explained by the smaller sample size in our prospective analysis and the habitual low baseline fiber intake that was not predictive of glycemic control 6 years later. This argument may further explain our observation of an inverse trend between fiber intake and poorly controlled HbA1c (> 7%) but only in the highest quintile of fiber intake in the T1D group. Poor glycemic control in T1D has been associated with excess mortality and cardiovascular disease (CVD) when compared to non-diabetic matched controls [20, 21], and few adults with T1D meet current glycemic control targets [20], thus necessitating additional preventive strategies such as dietary fiber intake in this high-risk population. Dietary fiber has been identified as a nutrient of public health concern and most of the US adults do not meet the recommendations of 38 g/day for adults [22]. We observed an intake of dietary fiber of less than half the recommendations in our cohort of individuals with and without T1D. These findings in adults may also be reflective of a continuum of poor dietary fiber intake observed in children with T1D [23], thus identifying a strong need to improve dietary fiber intake in early life in T1D populations.

Very few studies have been reported on the association of dietary fiber with glycemic control, especially in T1D. Our study findings agree with a few previously reported studies showing no prospective associations of dietary fiber with HbA1c in youths with T1D [9, 12], while, we observe similar findings of inverse cross-sectional association between dietary fiber and HbA1c in adults with T1D in the EURODIAB study [24], as well as in youths with T1D [25]. Our longitudinal findings differ from another report from the EURODIAB study in which baseline fiber intake revealed significant protective association against elevated HbA1c levels in a 6.8 year follow-up period [11]. In comparing our study with this previously reported study, we observe some differences, especially higher HbA1c at baseline and follow-up years, 8.25 ± 1.85% and 8.27 ± 1.44% (mean ± SD), respectively, in their study [11], vs. 7.9 ± 1.2% and 7.8 ± 1.12%, respectively, in our study. Also, our smaller sample size at the six-year follow-up (*n* = 990), when compared to this previous study (*n* = 1659) could explain the null findings in our longitudinal analysis. Further, there are numerous factors that impact changes in glycemic control over time, including adoption of new diabetes technology, such as the use of diabetes apps and remote glucose monitoring system [26] during the study period. Overall, the importance of increasing consumption of dietary fiber must be emphasized in the T1D population, based on the numerous health benefits of dietary fiber in delaying gastric transit time and improving postprandial glucose load, decreasing inflammation and cholesterol levels [27–29].

Our findings of the inverse association of dietary fiber with HbA1c in the non-diabetic controls agree with previous reports in such populations, and have implications for reducing risks associated with obesity, the metabolic syndrome and type 2 diabetes in the general population [30–33]. In addition to our main outcome of HbA1c, we also observed significant inverse correlations of dietary fiber with BMI, waist circumference, systolic and diastolic blood pressure, as well as serum cholesterol and triglycerides. These findings conform to reported studies on the protective associations of dietary fiber against obesity, the metabolic syndrome and elevated blood lipids [31, 32]. We also observed a moderately strong and significant inverse association between dietary fiber and total fat intake in the entire cohort, and this provides some evidence of unhealthy dietary patterns in T1D populations [34]. We have previously reported higher intake of total and saturated fats in the CACTI cohort that were associated with poor glycemic control [14]. Together with our current findings on fiber intake, selected nutrients, such as dietary fats, and dietary bioactive compounds may play an important role in glycemic control in T1D.

Our analyses have some limitations that must be considered during the interpretation of results. In the first place, nutritional exposure data at baseline dietary intakes from the FFQ relied on a retrospective self-report and, therefore, may have been prone to recall bias. Secondly, we did not have information on the distribution of soluble vs. insoluble fiber intake, food groups contributing to total fiber intake, as well as food bioactive compounds, such as resistant starch shown to be associated with improved glycemic mangement [9, 35, 36]. Thirdly, our study considered HbA1c as a marker of long-term glycemic control, and we did not have data on daily glucose measures to capture day-to-day glycemic fluctuations, which have recently been suggested as a strong predictor of overall glycemic control [37]. Future prospective cohort studies should thus examine associations of dietary fiber intake from different food groups, as well as different types of fiber using multiple biomarkers of glycemic control.

## Conclusions

In conclusion, our significant inverse association observed between dietary fiber intake and HbA1c in the model adjusted for age, sex and total calories, and diabetes duration for T1D at baseline visit (cross-sectional analysis) provides some evidence on the role of fiber intake in glycemic control, which is of importance in the management of T1D patients at a high risk of mortality from CVD. This association did not persist in models further adjusted for dietary macronutrients and plasma total cholesterol and triglycerides, and this may indicate that higher levels of fiber intake than the observed low habitual intakes are needed to counteract the positive associations of these variables with HbA1c. Overall, our adjusted correlation coefficients also revealed a significant inverse association of fiber intake with HbA1c in the entire cohort, as well as in T1D cases and non-diabetic controls. We used data from a well-characterized cohort of T1D patients as well as matched non-diabetic controls, thereby permitting generalizability of our data to these populations. Further research may explore whether overall dietary quality when adjusted for fiber intake is associated with glycemic control in participants with T1D or non-diabetic individuals with habitual low fiber intakes.
